# Thylakoid membrane reorganizations revealed by small-angle neutron scattering of *Monstera deliciosa* leaves associated with non-photochemical quenching

**DOI:** 10.1098/rsob.200144

**Published:** 2020-09-16

**Authors:** Renáta Ünnep, Suman Paul, Ottó Zsiros, László Kovács, Noémi K. Székely, Gábor Steinbach, Marie-Sousai Appavou, Lionel Porcar, Alfred R. Holzwarth, Győző Garab, Gergely Nagy

**Affiliations:** 1 Neutron Spectroscopy Department, Centre for Energy Research, H-1121 Budapest, Konkoly-Thege Miklós út 29-33, Hungary; 2 Laboratory for Neutron Scattering and Imaging, Paul Scherrer Institute, CH-5232 Villigen PSI, Switzerland; 3 Max-Planck-Institute for Chemical Energy Conversion, Stiftstr. 34-36, 45470 Mülheim a.d. Ruhr, Germany; 4 Biological Research Centre, Institute of Plant Biology, 6726 Szeged, Hungary; 5 Forschungszentrum Jülich GmbH, Jülich Centre for Neutron Science at MLZ, 85748 Garching, Germany; 6 Biological Research Centre, Institute of Biophysics, Temesvári körút 62, 6726 Szeged, Hungary; 7 Institut Laue-Langevin, BP 156, 38042 Grenoble Cedex 9, France; 8 Department of Physics, Faculty of Science, Ostrava University, Chittussiho 10, 710 00 Ostrava, Czech Republic; 9 European Spallation Source ESS ERIC, PO Box 176, 221 00 Lund, Sweden; 10 Institute for Solid State Physics and Optics, Wigner Research Centre for Physics, 1121 Budapest, Hungary

**Keywords:** chloroplast thylakoid membranes, grana, lamellar repeat distance, non-photochemical quenching, small-angle neutron scattering

## Abstract

Non-photochemical quenching (NPQ) is an important photoprotective mechanism in plants and algae. Although the process is extensively studied, little is known about its relationship with ultrastructural changes of the thylakoid membranes. In order to better understand this relationship, we studied the effects of illumination on the organization of thylakoid membranes in *Monstera deliciosa* leaves. This evergreen species is known to exhibit very large NPQ and to possess giant grana with dozens of stacked thylakoids. It is thus ideally suited for small-angle neutron scattering measurements (SANS)—a non-invasive technique, which is capable of providing spatially and statistically averaged information on the periodicity of the thylakoid membranes and their rapid reorganizations *in vivo*. We show that NPQ-inducing illumination causes a strong decrease in the periodic order of granum thylakoid membranes. Development of NPQ and light-induced ultrastructural changes, as well as the relaxation processes, follow similar kinetic patterns. Surprisingly, whereas NPQ is suppressed by diuron, it impedes only the relaxation of the structural changes and not its formation, suggesting that structural changes do not cause but enable NPQ. We also demonstrate that the diminishment of SANS peak does not originate from light-induced redistribution and reorientation of chloroplasts inside the cells.

## 1. Introduction

Oxygenic photosynthetic organisms protect themselves against photodamage under excess light conditions. In algae and higher plants, one of the most important photoprotective mechanisms is the non-photochemical quenching (NPQ) of the first singlet excited state of chlorophyll-a. *In vivo*, NPQ is not a homogeneous process [1–3]. Under most conditions its kinetics is dominated by the rapidly (approx. 30–60 s) induced and relaxing, ΔpH- or energy-dependent (qE) component, and a more slowly developing (2–10 min) and relaxing component. The underlying molecular mechanisms of NPQ are still debated [4–9]. In excess light, sustained acidification of the lumen is sensed by the PsbS protein in plants [10,11] and it also activates the xanthophyll cycle, leading to the conversion of violaxanthin to zeaxanthin [12–15]; both of these effects contribute to the generation of NPQ [9,16].

Numerous studies have shown that fine adjustments of the photosynthetic functions to different environmental conditions depend largely on the structural flexibility of thylakoid membranes [1,17–24]. Albeit details of the molecular mechanisms are still under debate, NPQ mechanisms have been thoroughly documented to be associated with structural changes in the thylakoid membranes. Following the protonation of PsbS, substantial reorganizations occur in the light-harvesting antenna system of photosystem II, including the aggregation of LHCII, its major light-harvesting antenna complex as well as its association with PsbS [25–31]. The functioning of the xanthophyll cycle (i.e. the de-epoxidation of violaxanthin to antheraxanthin and zeaxanthin) also involves significant reorganizations via the activity of the water-soluble, lipocalin-like enzyme violaxanthin de-epoxidase; the functioning of this enzyme requires the formation of a non-bilayer lipid phase [9,12,16–20,23,27,32–34]. In the sustained quenching component, qH, another lumenal lipocalin protein, LCNP, plays a central role [35]. Hence, in general, structural changes appear to be associated with NPQ at different levels of the structural complexity of thylakoid membranes: the molecular organization of the protein complexes, localization of the PsbS, oligomerization of LHCII, activity of water-soluble lipocalin (-like) proteins and the formation of non-bilayer lipid phase. In the light of these changes, one can pose the question how much, if at all, the entire thylakoid membrane system is involved in these reorganizations.

Although NPQ can be induced in isolated thylakoid membranes [36], the values of NPQ are much larger in intact systems [37]. Thus, preferably, measurements on intact leaves should be carried out in order to better understand the relation between qE and the chloroplast ultrastructure. This, optimally, requires the use of a non-invasive technique that is capable of providing time-resolved information on the organization of thylakoid membranes *in vivo*.

Small-angle neutron scattering (SANS) is a non-invasive experimental technique, which has proved to be a valuable tool in monitoring ultrastructural changes in biological systems [38–40]. It has been thoroughly documented that the multi-lamellar periodic thylakoid membrane systems of cyanobacteria, algal cells and intact leaves exhibit characteristic SANS profiles. SANS curves of thylakoid membranes with long-range order along the membrane normals exhibit characteristic peaks—so-called Bragg-diffraction peaks—whose characteristics provide information about the periodic arrangement of the membranes.

The Bragg diffraction peaks carry spatially and statistically averaged information on their repeat distances (RDs) [41–46]. The same studies have also revealed different reorganizations and changes in the membrane periodicity occurring on the time scale of minutes or shorter. These measurements have shown that thylakoid membrane systems should not be portrayed as merely providing scaffold for the protein complexes but the membrane system appears to play an active role in different regulatory mechanisms and must be considered to be highly dynamic. Indeed, in some cases, membrane reorganizations could be clearly linked to regulatory functions, such as the state transitions in the green alga *Chlamydomonas reinhardtii* [42], the aggregation-induced quenching in a desert-crust cyanobacterium [47], and changes in the RDs due to the presence or absence of ion channels in *Arabidopsis* leaves [48]. *In vitro* experiments have shown that gradually lowering the pH of the medium from 8.0 to 5.0 causes reversible RD- and mosaicity-changes in isolated plant thylakoid membranes [49]. These data strongly suggested correlation between NPQ and membrane reorganizations of grana. However, this hypothesis has not been tested further *in vivo* so far.

In the present study, in order to obtain real-time information on NPQ-associated thylakoid membrane reorganizations *in vivo*, we recorded time-resolved SANS profiles and chlorophyll-a fluorescence transients on *Monstera deliciosa* leaves. This climbing rainforest vine—being a secondary hemi-epiphyte—can survive both the low-light environment on the rainforest floor and the high-light environment of the sunlit canopy [50]. Though the widely used model plant, *Arabidopsis thaliana,* with a wide range of available mutant strains, should be an ideal candidate to study NPQ, its applicability for SANS measurements is somewhat limited due to its weak Bragg peak [48]. On the other hand, *M. deliciosa,* an evergreen model species, is an ideal system to our investigations, for two reasons. (i) The chloroplast of this species contains tall stacks of several dozens of firmly appressed granum thylakoids [51]. This renders these samples readily amenable for SANS experiments since the width of the Bragg diffraction peak depends on the number of the scattering bilayers (i.e. on the size of the lattice in the direction of the periodicity); higher and narrower Bragg peaks are expected with increasing number of bilayers [52]. (For the same reasons, wild-type and NPQ-mutant *Arabidopsis* leaves [10], for their small grana sizes and weak Bragg diffraction peaks [53], could not be used in the present study.) (ii) *Monstera deliciosa* leaves possess high capacity for ΔpH-dependent NPQ and greater expression of the PsbS protein compared to annuals [50,51,54]. In general, the highly dynamic structural flexibility of granum thylakoid membranes has been proposed to play important roles in different regulatory mechanisms [55].

Our SANS measurements have revealed that the periodic order of the grana are rapidly disrupted when *M. deliciosa* leaf segments are subjected to NPQ-inducing conditions. These dark-reversible ultrastructural changes occurred on a very similar time scale as the build-up and relaxation of NPQ. These results would suggest a causal correlation between the two phenomena. However, data obtained in the presence of the photosystem II inhibitor, diuron (DCMU, 3-(3,4-dichlorophenyl)-1,1-dimethylurea), allow only indirect correlation between the two. It is proposed that remodelling of the membrane system and, in general, the overall membrane reorganizations establish the conditions for the quenching of fluorescence (i.e. for the local action of effector molecules, such as zeaxanthin and PsbS, which are more directly responsible for the quenching of the excess excitation energy).

## 2. Material and methods

### 2.1. Plant cultivation

*Monstera deliciosa* plants were grown in large pots with gardening soil and fertilized with dilute liquid fertilizer (used for evergreens) once per week. The plants were provided 50–60 µmol photons m^−2^ s^−1^ light by an array of cool white fluorescent tubes with a 12 h light/12 h dark cycle in an indoor location where room temperature was maintained at 20 ± 2°C for the typical so-called ‘low-light-grown leaves'. So-called ‘high-light-grown leaves' were also measured. These were cultivated at a fully sun-exposed south-facing window and leaves were harvested in March and May when these plants were adapted to high light (up to 1600 µmol photons m^−2^ s^−1^ peak at noon). In all cases, full grown (at least 3–4 weeks old) and healthy leaves were cut and transported to Garching (Germany) and Grenoble (France) in moist condition in the dark. Unless stated otherwise, the figures shown in the present paper are from measurements on low-light-grown leaves; very similar data were obtained on high-light-grown leaves (see Results and electronic supplementary material).

### 2.2. Sample preparation

#### 2.2.1. Infiltration

In order to reduce the incoherent scattering from the sample and enhance the contrast between the membranes and the adjacent aqueous phases [56] for most samples infiltration was applied in order to replace part of the light-water content of the leaf with heavy water. In these samples before SANS measurements, about 1 cm × 4 cm segments were cut from the leaf, the epidermis of the lower (abaxial) side was gently scrubbed by sandpaper and the segments were then infiltrated in pure heavy water and put into quartz cuvette filled with heavy water. The measurements on both intact and infiltrated leaves were performed at ambient temperature.

#### 2.2.2. Magnetically oriented isolated thylakoid membranes

For the face- and edge-aligned SANS measurements, thylakoid membranes were isolated from freshly harvested 3 weeks-old pea leaves (*Pisum sativum*, Rajnai törpe) as described earlier [56]. Since these experiments serve only as a demonstration of the SANS signal of the differently oriented thylakoid membranes, pea leaves—providing well-characterized isolated thylakoid membranes—were used instead of *M. deliciosa* leaves. (Note that pea chloroplasts contain smaller grana and thus exhibit weaker Bragg diffraction peak.) The suspension of thylakoid membranes in D_2_O-containing medium (20 mM Tricine (p^2^H 7.6), 0.3 M NaCl, 5 mM KCl and 5 mM MgCl_2_) was prepared as described earlier [56].

### 2.3. SANS experiments

#### 2.3.1. Instrumentation and data acquisition

SANS measurements were performed on the KWS2 instrument of the Jülich Centre for Neutron Science (JCNS) at the Heinz Maier-Leibnitz Zentrum (MLZ), Garching, Germany and on the D11 instrument at the Insitut Laue-Langevin (ILL), Grenoble, France, where high-flux research reactors provide continuous neutron beam [57]. The produced neutrons are moderated in a cold source and monochromatized by velocity selector. The neutrons scattered from the sample are recorded by ^6^Li-scintillator (KWS2) [58] and ^3^He filled (D11) position-sensitive detectors. The sample-to-detector distance was set to 5.82 and 5.5 m, the wavelength was set to 4.53 and 6 Å at the instruments KWS2 and D11, respectively; the collimator distance was 8 m in both cases. With these settings, we could cover a *q*-range between about 0.0075 and 0.1 Å^−1^ (KWS2) and about 0.013 and 0.12 Å^−1^ (D11). In some samples, additional peaks were detected in the higher scattering vector range. The presence of these peaks can most likely be attributed to a very well defined periodic order of the granum thylakoid membranes and can be assigned to second- and third-order Bragg diffractions (electronic supplementary material, figure S1). The relatively low S/N ratios in the high-q range did not allow us to study the kinetics of the light-induced changes of the additional peaks. Thus, we confined our kinetic studies and data analysis on the first-order Bragg peak.

Non-infiltrated leaf segments were placed in empty quartz cuvettes. The D_2_O-infiltrated leaf pieces were placed in 1 mm (KWS2) and 2 mm (D11) quartz cuvettes filled with D_2_O. Samples were illuminated during the measurements with white or red light (greater than 650 nm cut-off glass filter), using Schott KL 2500 lamps and optical fibres; the light beams were close to parallel to the neutron beam.

All samples were measured at room temperature and the acquisition time was 1 min for the case of time-resolved measurements, while steady-state scattering curves were collected for 1–10 min. In total, 12 SANS experiments (three on non-infiltrated and nine on infiltrated leaf segments from a total of four different detached leaves) were performed under different actinic light intensities including the repetitions in JCNS and ILL. Rise and relaxation kinetics as well as the magnitude of changes varied from sample to sample but they showed very similar trends regarding the light-induced diminishment of the integrated intensity, and shrinkage, as well as the reversibility of the changes.

The isolated thylakoid membranes were filled in 1 mm quartz cuvettes and aligned in permanent magnets; for face- and edge-alignments, respectively, a magnet (Jasco) of 1.4T field strength and a home-built magnet of 0.7T were used. (Face- and edge-alignment of membranes refer to orientations of the membrane planes perpendicular and parallel to the direction of neutron beam, respectively.) In order to prevent scattering of the neutron beam from the magnets, cadmium sheet apertures were used.

#### 2.3.2. Data reduction and data treatment

The raw SANS data obtained at KWS2 and D11 were treated with the QtiKWS program [59] and the Graphical Reduction and Analysis SANS Program for Matlab—GRASP (developed by Charles Dewhurst, ILL), respectively. All data are normalized to the number of beam monitor counts, the background measured by boron carbide (KWS2) and cadmium (D11) was subtracted. The empty quartz cuvette scattering was subtracted from the sample scattering and the scattering data were corrected for the detector efficiency using standard procedures (precalibrated ‘plexiglas' at KWS2 and H_2_O at D11). The scattering intensities obtained from magnetically aligned isolated thylakoid membranes were radially averaged in two 45° sectors around each opposite Bragg diffraction peaks. Scattering data from leaves were radially averaged in 360°. The integrated peak intensity values were obtained by fitting a linear combination [60] of a constant, a power and a Gauss function on the radially averaged scattering curves

$$I(q)=I_{0}+A|q{|}^{p}+\frac{B}{w\sqrt{\pi /2}}{\text{e}}^{-2({\left(q-q^{*}\right)}^{2}/w^{2})},$$

where *I* is the scattered intensity, *I_0_* and *A* are constants, *p* is the parameter of the power function, *B* is the integrated intensity of the peak, q* is the centre position of the Bragg peak and *w* is related to the full width at half maximum of the diffraction peak; the repeat distance values were obtained by using RD = 2π/q*. In this article, the errors signify the uncertainty of the fitting (standard deviation).

### 2.4. NPQ measurements

The NPQ measurements were performed using a FluorPen FP100 (Photon System Instruments, Czech Republic) using the predefined protocol NPQ-2 (200 s of light exposure and 390 s of dark recovery). The measurements were carried out at several independent locations on a single leaf. The photon flux density of the blue saturating light pulses was 3000 µmol photons m^−2^ s^−1^ and that of the white actinic light was 1000 µmol photons m^−2^ s^−1^.

### 2.5. DCMU treatment

DCMU treatment was carried out by mild vacuum infiltration (three to four times, using a syringe) in a 300 µM (NPQ), or 400 µM (SANS) DCMU solution and was incubated for 1 h before the measurements. The epidermis of the lower (abaxial) side of the leaves was mildly rubbed with extra-fine sandpaper (ISO/FEPA Grit designation: P400, average particle diameter: 35.0 µm) before the DCMU treatment. At least 300 µM, DCMU was needed for achieving full PSII inhibition, as tested by fluorescence induction using a FluorPen instrument (FP 100 PSI). (High concentrations of DCMU were required most probably because of the poor penetration of this inhibitor into the thick leaf tissue.)

## 3. Results and discussion

### 3.1. Light-induced thylakoid membrane reorganizations associated with NPQ

**Figure 1.**
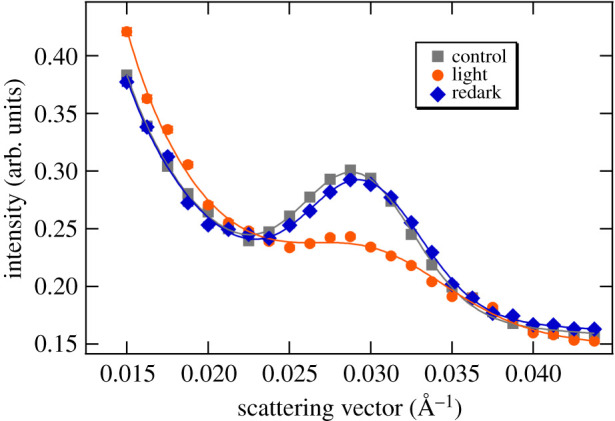
Typical SANS profiles of a non-infiltrated dark-adapted leaf segment (control) of *M. deliciosa*, and upon its 8 min illumination with white light of 300 µmol photons m^−2^ s^−1^ (light) and a consecutive 30 min dark period (redark). The profiles represent 5 min averaging at the end of each period; solid lines are fitted curves. The acquisition time was 1 min; consecutive profiles were then averaged to improve the relatively low S/N ratio due to the presence of light water in the leaf tissue. (The measurements were carried out on KWS2.)

We performed initial experiments on *M. deliciosa* leaf sections without infiltration and perceived a strong Bragg peak at 0.029 Å^−1^, corresponding to a lamellar RD of about 215 Å, and significant variations in the SANS profiles (ΔSANS) upon illumination of the leaf segments (figure 1). The illumination conditions were set to induce NPQ (see below).

Upon illumination, the most dominant alteration of the scattering peak was the decrease of the integrated intensity of the Bragg peak (to *ca* 65% of the dark control); the variation was largely reversible upon a 30 min dark re-adaptation (to approx. 95% of the original intensity). We also observed a slight shift in the peak position (from 0.02924 ± 0.00004 Å^−1^ to 0.0302 ± 0.0001 Å^−1^), corresponding to an RD decrease from about 215 ± 1 Å to 208 ± 1 Å; this also tended to reverse during the consecutive dark-adaptation period (q* = 0.02965 ± 0.00005 Å^−1^, corresponding to 212 ± 1 Å). We also observed an increase in the full width at half maximum (FWHM) of the Bragg peak from 0.0081 ± 0.0001 Å^−1^ (dark control) to 0.01095 ± 0.0004 Å^−1^ (light-adapted state), which was reversible upon consecutive dark re-adaptation (0.0081 ± 0.0001 Å^−1^). Similar light-induced, dark-reversible reorganizations have earlier been observed on isolated spinach thylakoid membranes subjected to illumination [60], which were attributed to a reversible decrease in the long-range order of the thylakoid membranes.

**Figure 2.**
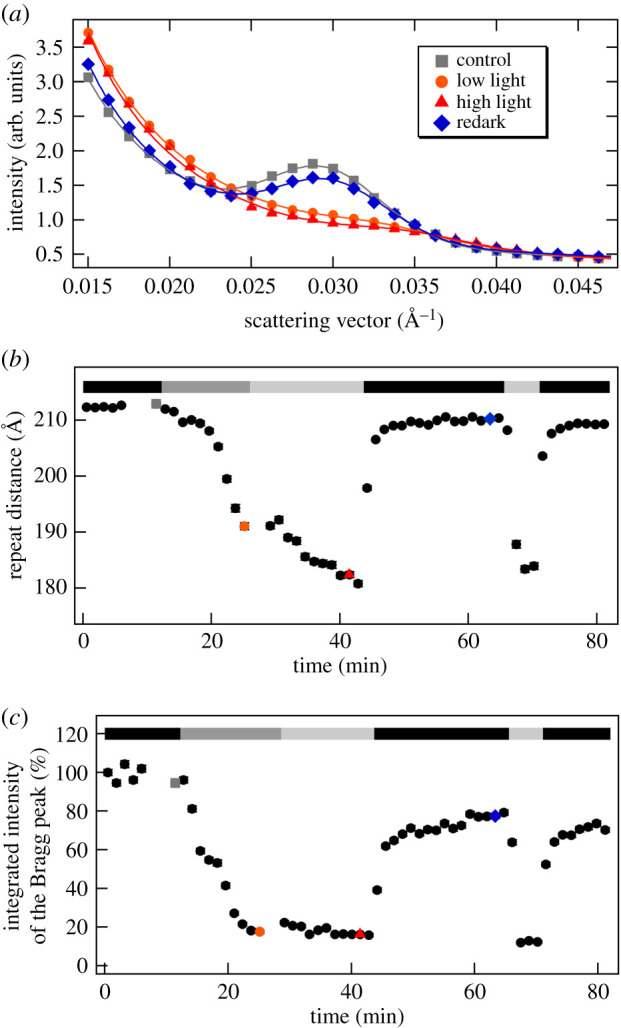
SANS profiles of D_2_O-infiltrated *M. deliciosa* leaf segments and kinetic traces during two dark-light-redark cycles. (*a*) Radially averaged SANS curves recorded in the dark, and under low light (300 µmol photons m^−2^ s^−1^) and high light (800 µmol photons m^−2^ s^−1^) conditions, followed by a dark period (redark); solid lines represent the fitted curves. (*b*) and (*c*), respectively, show variations of the fitted RD and integrated intensity values of the Bragg peak, relative to the dark control; the black, dark grey and light grey bars indicate dark conditions and low- and high-light illuminations, respectively; grey, orange, red and blue dots refer to the measured curves in (*a*). (Measured on KWS2.)

With the improved contrast, and thus better S/N, in the D_2_O-infiltrated leaf segments, ΔSANS could be observed with a time resolution of 1 min, allowing the monitoring of the kinetics of reorganizations occurring on the time scale of several minutes to tens of minutes (figure 2). It can be seen that both the shift in the RD to lower values (figure 2*b*) and the decrease in the integrated intensity (figure 2*c*) of the Bragg peak displayed a fast recovery in the dark period after the illumination. It is to be noted here that, as also follows from a comparison of figures 1 and 2, the position of the Bragg diffraction peak and thus the RD values (215 ± 1 and 212 ± 1 Å, respectively) were largely invariant on the infiltration of leaves with D_2_O (cf. also [56]). On the other hand, the magnitude of the light-induced changes appeared to increase after infiltration, which can be explained by the better penetration of light after infiltration. The non-infiltrated leaves were virtually non-transparent, leading to very large gradients in the transverse distribution of the actinic light [61]. By contrast, neutrons scattered in the forward direction carry spatially averaged information for the entire volume irradiated by the neutron beam, irrespective of the inhomogeneity of illumination and the attenuated intensity of the actinic light. With this constraint, the phenomena observed in D_2_O-infiltrated and non-infiltrated leaf sections are in good agreement with each other.

Also shown in figure 2, the structural flexibility of the membrane system was retained during repeated light–dark cycles. However, it must be noted that the recovery of the periodic order of the multi-lamellar membrane system, on the fast, few-minutes time scale, remained incomplete; the relaxation of ΔSANS evidently contained slower processes. It is also interesting to note that the time courses of light-induced variations in the RD and the integrated intensity were not identical, at least when starting with dark-adapted sample (cf. figure 2*b*,*c*). The integrated intensity of the Bragg peak decreased to as low as 20% of its original value after less than 10 min of low-intensity (300 µmol photons m^−2^ s^−1^) white-light illumination and no further decrease was induced by the consecutive illumination with an increased light intensity (800 µmol photons m^−2^ s^−1^). By contrast, the peak position appeared to further shift towards higher *q*-values, reaching a value corresponding to an RD of about 180 Å. (Similar time-resolved light-induced dark-reversible diminishment in the intensity of Bragg-diffraction peak of grana, accompanied by a decrease in the calculated RD, using the q* of the remaining peak, has earlier been observed on isolated tobacco thylakoids, using SANS [56]. Light-induced, dark-reversible shrinkage of granum thylakoid membranes was first observed by Murakami & Packer in 1970 [62], via analysing electron microscopy images of isolated dark-adapted and illuminated isolated thylakoid membranes.) It is interesting to note that during the repeated dark–light cycle the kinetics of the two parameters became more similar; these kinetic traces were dominated by the rapid variations both in the measured RDs and the integrated intensities (figure 2).

It is interesting to note here that the RD values in low-light grown and high-light grown leaves under dark adaptation were essentially identical (electronic supplementary material, table S1). This is at variance with transmission electron microscopy (TEM) data [51], which revealed larger RD in the dark-adapted high-light grown leaves compared to that of the low-light grown leaves (214 and 192 Å, respectively, as calculated from the number of granum thylakoids per unit distance published in [51]). Also, kinetics of ΔSANS revealed no significant difference between the two types of leaves (electronic supplementary material, figure S2). In contrast to a substantial swelling (from 214 Å to 265 Å) upon illumination of the high-light grown leaves with 1500 µmol photons m^−2^ s^−1^ light (TEM data), ΔSANS under comparable conditions displayed shrinkage, similar to low-light grown leaves, accompanied by the diminishment of the Bragg peak (electronic supplementary material, figure S3). The lack of differences between the low-light grown and high-light grown samples, both with regard to the dark RD values and the light-induced changes, can most likely be explained by the condition that the detached leaves were kept in darkness or dim light for 2–3 days before the SANS measurements (see Material and methods). This explains that SANS and ΔSANS features of high-light-grown leaves resembled closely to those of the low-light grown leaves. Also worth noting that with 2000 µmol photons m^−2^ s^−1^ illumination the scattering changes occurred considerably more rapidly than with lower light intensities (cf. with electronic supplementary material, figure S3 and figure 2).

The observed light-induced variations in the scattering curves can be attributed to two probably independent processes: (i) the substantial decrease of the integrated intensity of the Bragg peak shows that illumination disrupts the long-range periodic order of the thylakoid membranes; (ii) this process, as indicated by the RD decrease, is associated with a shrinkage of the membrane system.

As to these reorganizations, it is unclear if the remodelling of grana occurs homogeneously or if loosely and tightly stacked regions display different patterns. The strength of stacking in the grana has been shown to have inhomogeneous nature, being stronger in the middle of the granum and weaker towards the margins [63–65]. Hence, it cannot be ruled out that the observed variations in the scattering signal reflect a selective loss of the Bragg diffraction peak of a subpopulation of loosely stacked membranes, which display lower q* (higher RD) values. This assumption is supported by the fact that loosely stacked regions, and/or grana margins, in general, for their wider D_2_O-enriched aqueous phases, are expected to exhibit stronger scattering signal. Vice versa, penetration of D_2_O into tightly stacked inter-thylakoidal regions might be more restricted, which—together with the influence of the protein segments protruding into the inter-thylakoidal space—result in weaker contrast. The differential contributions of loosely and tightly stacked regions might, however, be counteracted by their different structural flexibilities—loose stacking might be more prone to undulations, weakening and broadening the Bragg diffraction. Conversely, tight stacking is expected to result in sharper Bragg peak. The observation that the decrease in the overall intensity of the Bragg peak is accompanied by an overall broadening strongly argues against the possibility that the reorganizations are confined to the loosely stacked regions of the grana, such as via an unstacking in the marginal regions, with the core of the grana unaffected. It seems thus more likely that the membrane reorganizations are extended over the entire granum and involve also—or are even dominated by—the tightly stacked regions. These data also support, at least in part, the notion that dissociation of LHCII from PSII and its aggregation under NPQ conditions in spinach chloroplasts is related to the stacking of membranes [9,27]. Taking together these observations and considerations, our data clearly show that NPQ-inducing illumination of *M. deliciosa* leaf segments leads to an overall pronounced remodelling of the thylakoid membrane system.

In general, TEM, beside its considerably lower sensitivity to small RD changes and subtle lamellar disorder, is evidently not suitable for monitoring the kinetics of membrane reorganizations in a leaf section. The fixation procedure takes too long for kinetic measurements. Glutaraldehyde penetrates tissues slowly (1 mm h^−1^), osmium tetroxide is even slower (0.5 mm h^−1^) [66]. The slow penetration of fixatives into a (quite thick) Monstera leaf would pose serious limitations both regarding the time-resolution and the homogeneity of sample. At the same time, by comparing TEM and SANS data, for static cases, we have earlier confirmed that the information derived from the two techniques are in good agreement with each other—albeit some artefacts and biases on both sides cannot be ruled out [56]. Also, as pointed out above, TEM [51] in a low-light-grown sample revealed similar shrinkage as our ΔSANS measurements.

The observed reorganizations on the mesoscopic scale, reflected by ΔSANS, are probably associated with microscopic structural changes, which, acting together, set the stage for NPQ-effector proteins and molecules (e.g. the PsbS and zeaxanthin) [9,10,16,67–70]. In excess light, sustained acidification of the lumen is sensed by PsbS protein in plants and triggers the qE through protonation of PSII proteins, it also activates the xanthophyll cycle [11,71,72]. In general, low pH and the light-induced transmembrane ΔpH have been shown to induce structural changes at different levels of structural complexity, at the microscopic levels affecting the distribution of protein complexes (for reviews see [22,73], the lipid phases [74,75], and assemblies at higher levels of the membrane organization [17,18,20,21,49,76–78]. While structural changes appear to be ubiquitous in oxygenic photosynthetic organisms, NPQ is not. One of the clear examples is the PAL mutant of *Synechocystis*, which has been shown to respond to illumination by structural changes similar to the wild-type, but—in the absence of phycobilisome—exhibit no NPQ [41,44]. Hence, it is not obvious if the light-induced overall membrane reorganizations *in vivo*, reflected by ΔSANS, are directly correlated with NPQ.

**Figure 3.**
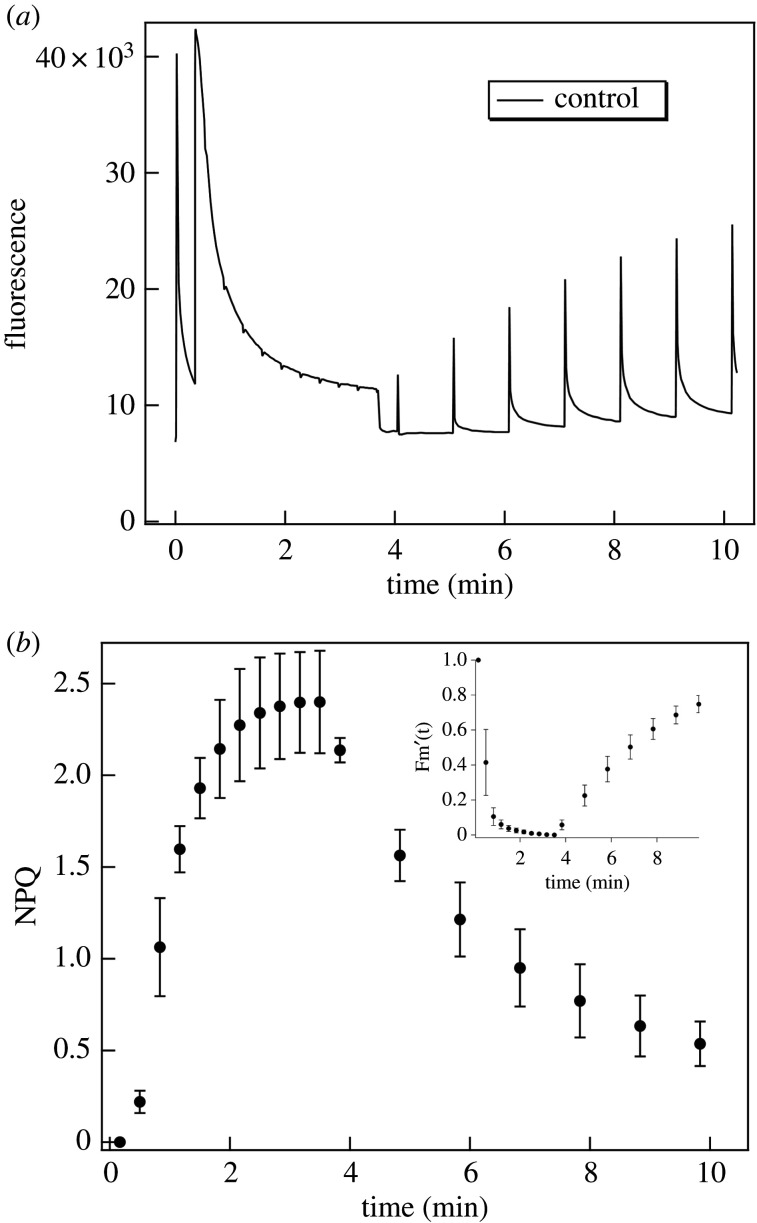
(*a*) Typical fluorescence kinetic trace and (*b*) averaged calculated NPQ kinetics of untreated *M. deliciosa* leaf segments. Inset: averaged calculated Fm'(t) versus time. The photon flux density of the actinic white light was 1000 µmol photons m^−2^ s^−1^; data points in (*b*), calculated mean values ± s.d., *n* = 3; (for further details, see text).

In order to investigate the putative correlation between NPQ and the light-induced ΔSANS, we carried out NPQ measurements under similar conditions as in the ΔSANS experiments. (figure 3). It can be seen that ΔSANS and NPQ occur with similar time courses. Both NPQ and ΔSANS develop on a fast time scale, with halftimes which can be faster than a minute. NPQ with 1000 µmol photons m^−2^ s^−1^ almost fully developed in less than a min. The halftime of Fm' decay (cf. [79].) was 0.41 ± 0.09 min (*n* = 3). The relaxation of NPQ was incomplete in the 10 min period of measurement. The recovery phase of Fm'(t) contained an exponential rise, with a halftime of 3.3 ± 0.5 min. The averaged Fm'(t) is shown as inset in figure 3*b*.

Although the time resolution of ΔSANS measurements, with 1 min acquisition times, does not allow a quantitative comparison with NPQ (Fm') kinetics, it can be seen that light-induced membrane reorganizations occur on a very similar time scale. Furthermore, the rise and decay kinetics of the structural changes accelerate upon repeated excitation (figure 2); a similar tendency is known for the kinetics of NPQ.

Taken together, the light-induced and dark relaxation kinetics of ΔSANS and NPQ are in good agreement with each other. However, we must stress that the experimental conditions in the two types of measurements are not identical—mainly because in leaves there are strong light gradients [80]. In our NPQ experiments, we used the commonly applied geometry of front-side excitation and detection. In a thick leaf, such as that of Monstera, the fluorescence signal is predominantly collected from a thin, highly illuminated layer of cells near the leaf surface. By contrast, in ΔSANS, all the detected scattered neutrons traverse the sample and—due to the low attenuation of the neutron beam inside the tissue—provide structural information almost uniformly for the entire volume irradiated by the neutron beam (i.e. also including those layers which are only weakly illuminated after traversing the strongly absorbing strata). These factors hinder a more quantitative comparison of ΔSANS and NPQ kinetics. Nevertheless, the data above would suggest a close correlation between the two phenomena which, however, as will be shown below, appears to be incomplete or indirect.

### 3.2. Light-induced thylakoid membrane reorganizations and NPQ in the presence of DCMU

**Figure 4.**
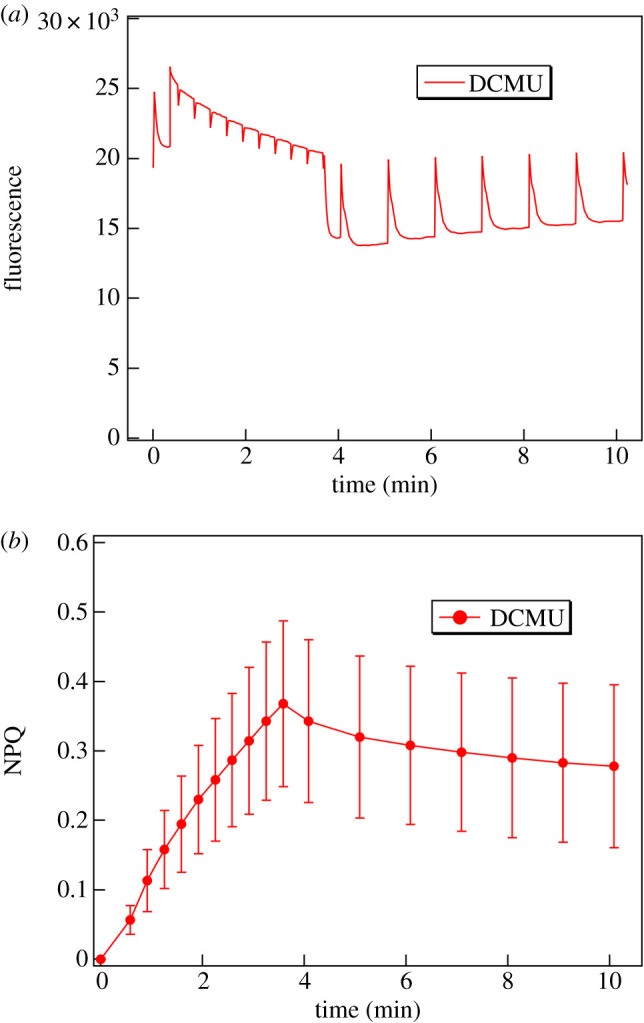
(*a*) Typical fluorescence kinetic traces and (*b*) averages of the calculated NPQ kinetics of DCMU-treated *M. deliciosa* leaf segments. The fluorescence induction was induced by white light of 1000 µmol photons m^−2^ s^−1^ (data points in (*b*), calculated mean values ± s.d., *n* = 6).

DCMU, which inhibits the electron transfer between the primary and secondary quinone electron acceptors of photosystem II, also inhibits the linear electron transport-dependent build-up of the pH gradient and, as a consequence, the NPQ is also inhibited. In accordance with expectations, DCMU substantially slowed down the development of NPQ and largely diminished its magnitude (figure 4), but did not entirely prevent it, possibly because of a cyclic PSI activity. The maximum value of the averaged NPQ of DCMU-treated leaf segments was only around 15% of the untreated samples; also, the recovery of fluorescence yield was essentially absent.

**Figure 5.**
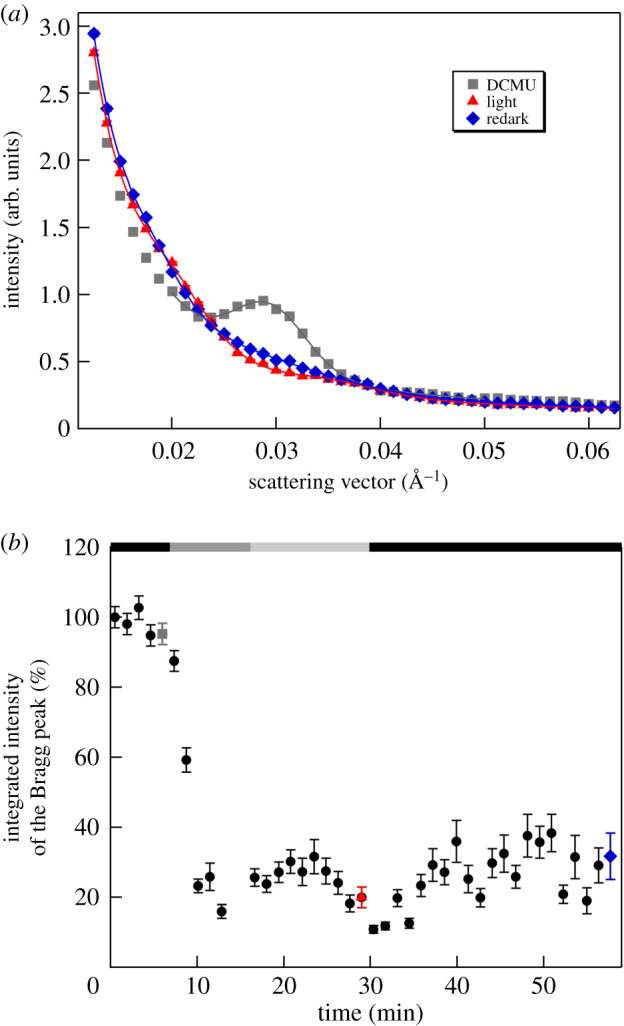
(*a*) SANS profiles of a D_2_O-infiltrated DCMU-treated *M. deliciosa* leaf segment in the dark, after their illumination and in redark; the samples were dark-adapted for 5 min, illuminated with white light of 300 µmol photons m^−2^ s^−1^ and 800 µmol photons m^−2^ s^−1^ and in the dark after the illumination period; the lines represent the fitted curves. (*b*) Integrated intensity of the Bragg peak versus time; the black, dark grey and light grey bars indicate dark conditions, 300 µmol photons m^−2^ s^−1^ and 800 µmol photons m^−2^ s^−1^ illuminations, respectively; grey, red and blue dots indicate the time of the measured curves. (Measured on KWS2.)

In contrast to the development of NPQ, the light-induced reorganizations of thylakoid membranes were not inhibited by DCMU (figure 5), suggesting different driving forces behind the two processes. The integrated peak intensity upon illumination decreased to about 20–30% of that in the dark, a value similar to that in the control (cf. figure 2). In contrast to the untreated sample, however, this diminishment in the Bragg peak in the DCMU-treated sample remained largely irreversible. While presently no explanation can be offered for this finding, we would like to note that it is in perfect agreement with the following similar observations. (i) By using SANS it has been shown that the light-induced swelling of the thylakoid membranes in the diatom *Phaeodactilum tricornutum* is fully reversible in the absence of DCMU [41], those induced in the presence of DCMU—although they are of comparable magnitude and rise kinetics—are irreversible on the same time scale [81]. (ii) A recent study, using neutron spin-echo, has revealed that the mechanical properties of *Synechocystis* thylakoid membranes are affected in a complex manner by DCMU; in particular, the membranes of the DCMU-treated cells appeared less flexible compared to the native membranes during the dark phase [82].

### 3.3. Light-induced chloroplast-movement related variations in the SANS profiles

When exposed to high light, chloroplasts in leaves can respond with a light-avoidance movement on the time scale of several minutes—they move to the side walls of cells, parallel to the illumination direction. This is a universal regulatory mechanism in vascular plants and, with the exception of mosses and ferns, it is governed by blue-light receptors [83]. In order to maximize the light capture, chloroplasts arrange along the upper and lower cell walls with their membrane planes preferentially perpendicular to the incident rays. Contrary to this arrangement, under excess light, they move to the side walls parallel to the rays, i.e. showing their edge to the illuminating beam and minimizing their absorbance and create shield for each other [84,85]. Such realignments might occur to a limited extent in the given geometry, illuminating the leaf segments at a narrow angle (almost parallel with the neutron beam) with white light. In general, realignments, by modulating the number of membranes in Bragg diffracting orientation, can significantly change the intensity of the Bragg peak. In order to test if realignments of this kind play any role in the observed light-induced changes in the SANS profiles, we performed experiments on magnetically aligned isolated thylakoid membranes, and also tested the effect of red-light induced SANS changes on *M. deliciosa* leaf segments.

**Figure 6.**
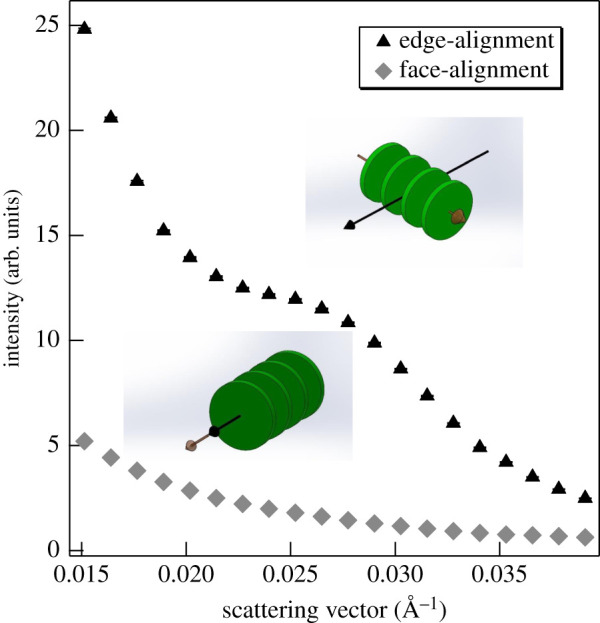
SANS profiles of isolated pea thylakoid membranes in face- and edge-aligned orientations, as indicated. The membranes are represented by flat green discs; black full arrowhead, direction of the neutron beam; brown empty arrowhead, magnetic vector. (Measured on KWS2, integration times, 2.5 (edge-aligned) and 5 min (face-aligned).)

As shown in figure 6, edge-aligned thylakoid membranes display a well-defined Bragg peak. This is in agreement with our earlier data, in which it has also been shown that the intensity of the Bragg peak could significantly be enhanced by aligning isolated thylakoid membranes in a magnetic field perpendicular to the neutron beam [60]. By contrast, face-aligned thylakoid membranes (in a magnetic field parallel to the neutron beam) exhibit no sizeable Bragg peak (figure 6).

Taking these data and the kinetics of SANS changes into account, it appears highly unlikely that the light-induced diminishment of the Bragg peak arises from chloroplast movements inside the cells. (i) The movements (turning away of chloroplasts from high light), in the given geometry, are expected to increase rather than to decrease the SANS amplitude of the Bragg peak. At low light condition, the chloroplasts are expected to move in face-aligned (zero Bragg diffraction) position, yielding maximum light absorption, while at high light intensity they are positioned away from strong light—in edge-aligned (maximum Bragg diffraction) position. (ii) The observed changes in SANS are much faster compared to what is expected for chloroplast movements, which requires the accumulation of chloroplast (cp) actin filaments to the leading edge of chloroplast. The relocalization of cp-actin filament can only occur in a few minutes [83,86,87], while the SANS changes can essentially be completed in less than 2 min (electronic supplementary material, figure S3).

**Figure 7.**
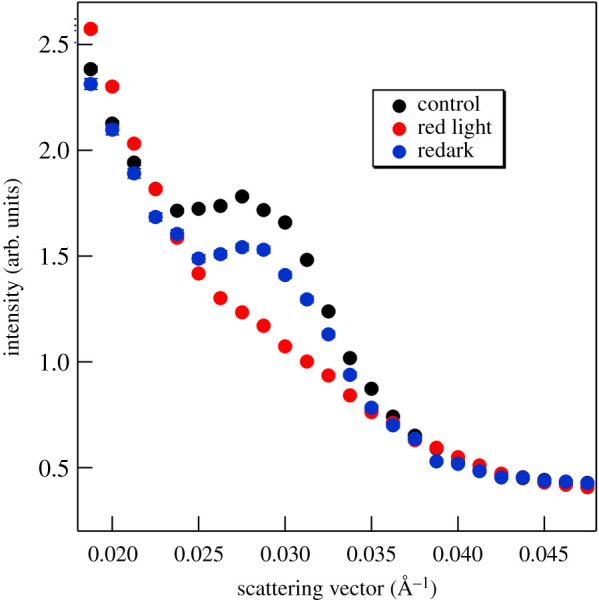
Effect of red-light illumination of 280 µmol photons m^−2^ s^−1^ on the SANS profile of a D_2_O-infiltrated *M. deliciosa* leaf segment. The light and redark profiles were recorded for 1 min at the end of the 9 min illumination and 10 min redark periods, respectively. (Measured on KWS2.)

In order to further substantiate this conclusion, we show that the reversible diminishment of the Bragg peak can also be induced by relatively weak (280 µmol photons m^−2^ s^−1^) red light (figure 7). The pattern of the chloroplast distribution depends on the light intensity; more precisely, on the intensity of UV-A/blue light [88], because only the blue light perceived by phototropins is active in the terrestrial angiosperms [85,89,90]. Hence, it can be concluded that the observed strong-light induced decrease of the Bragg peak cannot arise from light-induced chloroplast realignments in the cells.

**Figure 8.**
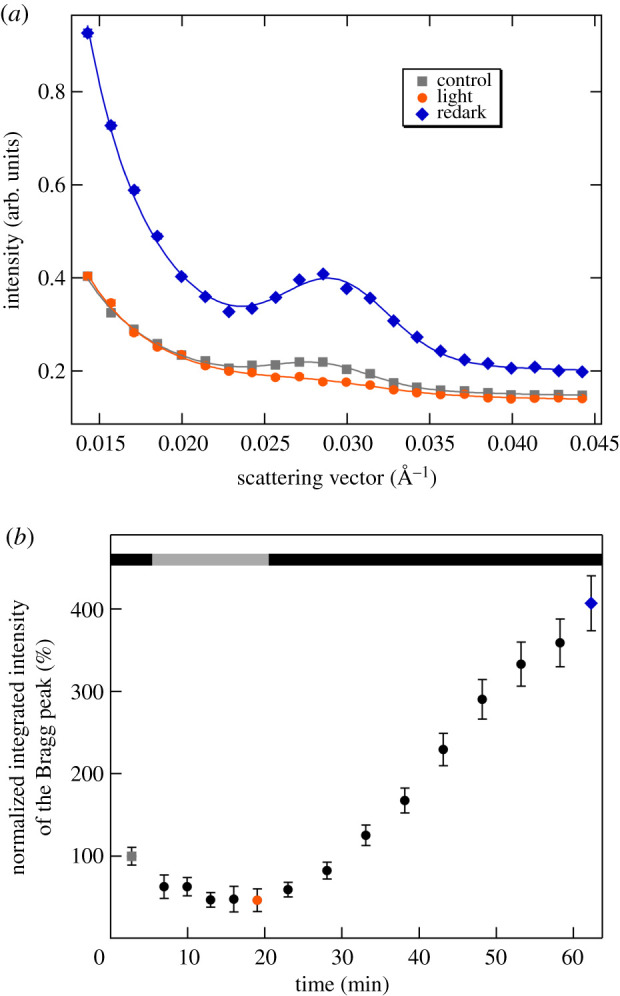
Effect of a dark-light-redark cycle on the SANS profile of a D_2_O-infiltrated M. deliciosa leaf segment. (*a*) Radially averaged SANS curves in the dark, following the illumination of the leaf section with white light of 400 µmol photons m^−2^ s^−1^ for 15 min and then kept in dark for 40 min; the lines represent the fitted curves. (*b*) Variations in the integrated intensity of the Bragg peak during the illumination and redark periods; black and grey bars indicate the dark and the illumination periods, respectively; grey, orange and blue dots refer to the measured curves of the same colour-codes. (Measured on D11.)

Interestingly, in some experiments with prolonged illumination and redark periods, we also observed a substantially increased intensity of the Bragg diffraction in the redark sample compared to the dark control before the illumination (figure 8). These changes are attributed to relocation and reorientation of chloroplasts rather than membrane remodelling: (i) they occurred on a time scale much longer than the membrane reorganizations, and (ii) no significant changes could be discerned either in the peak position or the half-bandwidth of the Bragg diffraction (data not shown). Although the experimental conditions and physiological state of leaves which reproducibly lead to similar increments remain to be identified, we think that this phenomenon deserves mentioning. It strongly suggests that SANS is capable of monitoring chloroplast reorientations inside plant cells and intact leaves.

## 4. Conclusion

By employing the non-invasive technique of small-angle neutron scattering on *M. deliciosa* leaf segments, we have revealed light-induced remodelling of the thylakoid membrane system under NPQ-inducing illumination conditions. These measurements revealed a substantial diminishment of the long-range, periodic order of granum thylakoid membranes; these changes were almost fully and rapidly reversible in the dark. Comparison of kinetic NPQ and SANS measurements, performed under comparable conditions, pointed towards a close correlation between NPQ and the reorganizations of the membrane system. However, experiments on NPQ-impaired, DCMU-treated leaf segments, exhibiting similar albeit irreversible light-induced SANS changes, have shown that the two processes are only indirectly linked to each other. The membrane reorganizations on the mesoscopic scale are proposed to enable NPQ by promoting the action of effector molecules which, on the microscopic scale, lead to the quenching of the excess excitation energy.

## Supplementary Material

Supplementary Information

## Data accessibility

The data that support the findings of this study are available from the corresponding authors, G.G. and G.N., upon reasonable request.
