# Supplementary material for: Thylakoid membrane reorganizations revealed by small-angle neutron scattering of Monstera deliciosa leaves associated with non-photochemical quenching
Source: Open Biol. 2020 Sep 16;10(9):200144. doi: 10.1098/rsob.200144 (PMC7536078; doi:10.1098/rsob.200144)
Supplement: Supplementary Information [file rsob200144supp1.docx]

**Supplementary Information**

**(Open Biology, DOI: 10.1098/rsob.20200144)**

**Thylakoid membrane reorganizations revealed by small-angle neutron scattering of *Monstera deliciosa* leaves associated with non-photochemical quenching**

**Renáta Ünnep^1,2^, Suman Paul^3#^, Ottó Zsiros^4^, László Kovács^4^, Noémi K. Székely^5^, Gábor Steinbach^6^, Marie-Sousai Appavou^5^, Lionel Porcar^7^, Alfred R. Holzwarth^3^, Győző Garab^4,8*^, Gergely Nagy^2,9,10,&*^**

^1^Neutron Spectroscopy Department, Centre for Energy Research, H-1121 Budapest, Konkoly-Thege Miklós út 29-33, Hungary

^2^Laboratory for Neutron Scattering and Imaging, Paul Scherrer Institute, CH-5232 Villigen PSI, Switzerland

^3^Max-Planck-Institute for Chemical Energy Conversion, Stiftstr. 34-36, D-45470 Mülheim a.d. Ruhr, Germany

^4^Biological Research Centre, Institute of Plant Biology, H-6726 Szeged, Hungary

^5^Forschungszentrum Jülich GmbH, Jülich Centre for Neutron Science at MLZ, D-85748 Garching, Germany

^6^Biological Research Centre, Institute of Biophysics, Temesvári körút 62, H-6726 Szeged, Hungary

^7^Institut Laue-Langevin, BP 156, F-38042, Grenoble Cedex 9, France

^8^Department of Physics, Faculty of Science, Ostrava University, Chittussiho 10, CZ-710 00 Ostrava, Czech Republic

^9^European Spallation Source ESS ERIC, P. O. Box 176, SE-221 00 Lund, Sweden

^10^Institute for Solid State Physics and Optics, Wigner Research Centre for Physics, H-1121 Budapest, Hungary

^#^Present address: Department of Biochemistry and Biophysics, Stockholm University, SE-10691 Stockholm, Sweden

^&^Present address: Neutron Scattering Division, Oak Ridge National Laboratory, Oak Ridge, TN 37830, USA

*Corresponding authors:

Neutron Scattering Division, Oak Ridge National Laboratory, Oak Ridge, TN 37830, USA

Telephone: +1 865 341 0482

e-mail: gergely.nagy.risp@gmail.com, nagyg@ornl.gov

ORCID number: 0000-0003-2742-0198

Biological Research Centre, Institute of Plant Biology, 6726 Szeged, Hungary

Telephone: +36 62 599 709

e-mail: [garab.gyozo@brc.hu](mailto:garab.gyozo@brc.hu)

ORCID number: 0000-0002-3869-9959


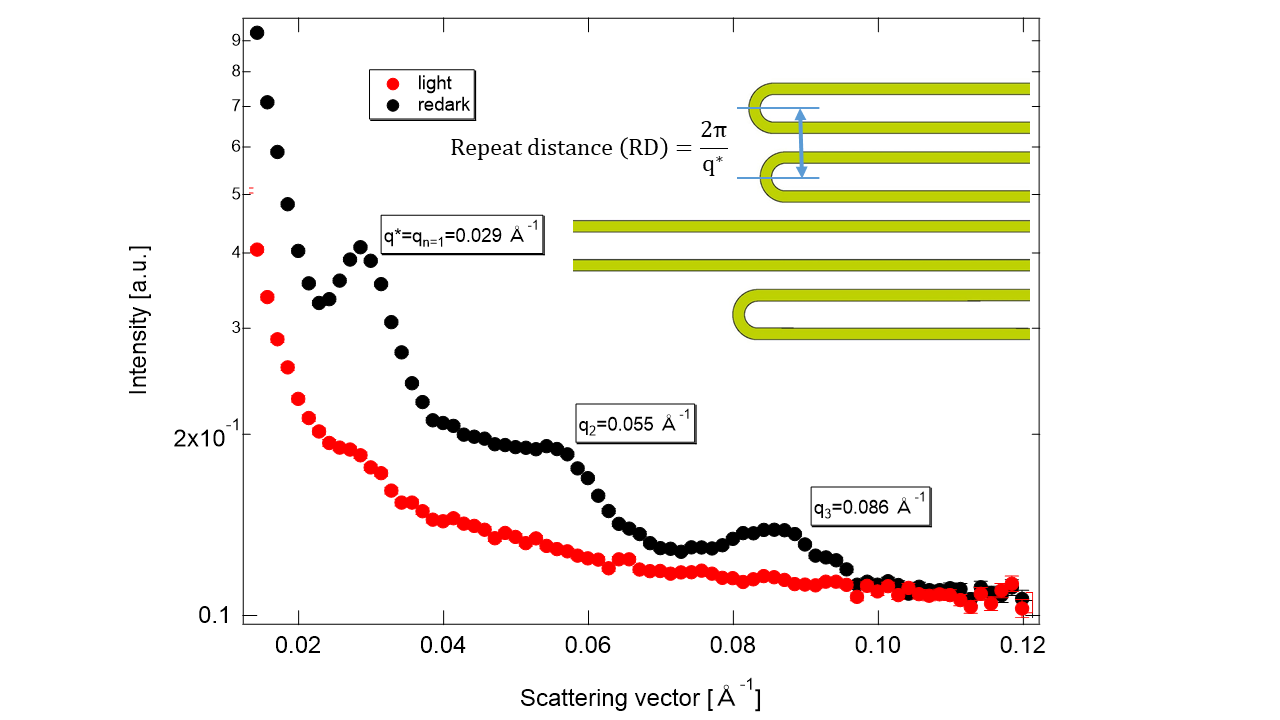


**Supplementary Figure 1.** Radially averaged SANS curves, scattering intensity as a function of the scattering vector q, of a D_2_O-infiltrated *M. deliciosa* leaf segment illuminated with white light of 400 µmol photons m^-2^ s^-1^ for 15 min and then kept in dark for 40 min (same measurement as in Figure 8, showing broader scattering-vector range). To improve the S/N ratio, the curves represent averages of 10 consecutive scattering profiles, with 1-min acquisition times each, at the end of illumination and redark periods. The first peak, at 0.029 Å^−1^, is the first-order Bragg diffraction peak, marked as q*=q_n=1_; the peaks at higher q values most likely originate from second-order (q_2_) and third-order (q_3_) diffractions. Inset: schematic drawing of stacked thylakoid membranes with a periodicity characterized by a repeat distance (RD), which can be calculated from the first-order Bragg diffraction peak (q*). (Measured on D11.) Similar data on dark-adapted spinach leaves, displaying three peaks, have earlier been published by Ünnep et al. (2014).

Formulation and implementation of a full mathematical model of the scattering profile would require the determination of the form factor of granum thylakoid membranes, following a procedure similar to that published recently for cyanobacterial thylakoids (Jakubauskas et al. 2019). This could, in principle, be performed via establishing the scattering density profile of the unit cell of grana, including the lipophylic phase and the lumenal and interthylakoidal aqueous phases, which are densely packed with proteins of different compositions. Such a model should also include the known polydispersity of granum stacking. Although the construction of a mathematical model of this kind is beyond the scope of the present study, we can safely conclude that illumination of the thylakoid membranes in vivo disrupts the periodic order of the thylakoid membranes, which, at the same time, most likely also affects the long-range order of the protein arrays. (For further discussion see main text.)

**Supplementary Figure 2.** Light-induced decrease of the integrated intensity of the Bragg-diffraction peak of low-light grown and high-light grown *M. deliciosa* leaves relative to the corresponding intensities before the illumination of dark-adapted D_2_O-infiltrated leaf segments with white light of 650 µmol photons m^-2^ s^-1^. Note that the decay kinetics is faster than with the lower intensity (300 µmol photons m^-2^ s^-1^) illumination (Figure 2B). Instrument (D11) settings: sample-to-detector distance, 8 m; wavelength, 6 Å; collimation distance, 8 m.

**Supplementary Figure 3.** SANS profiles of a D_2_O-infiltrated leaf segment of high-light grown *M. deliciosa* in dark adapted state (control), and upon 4-min long illumination with white light of 2000 µmol photons m^-2^ s^-1^ and a consecutive 12 min dark period (redark). The acquisition time of each profile was 1 min; 1.5 and 3.5 min are the profiles obtained from the second and fourth 1-min long measurements; control and redark profiles were obtained after 7 and 4 min averaging at the end of dark and redark periods, respectively. These data show that the membrane reorganizations are very rapid, and essentially completed in less than 2 min; and the changes are almost fully reversible. In further experiments (data not shown) we have observed that the reversibility of the structural changes was largely retained also after longer (15 min) illumination period with white light of 2000 µmol photons m^-2^ s^-1^ photon flux density. Instrument (D11) settings: sample-to-detector distance, 8 m; wavelength, 6 Å; collimation distance, 8 m.

**Supplementary Table 1.** Comparison of the RD values of low-light grown and high-light grown *M. deliciosa* D_2_O-infiltrated leaf segments obtained from two independent batches of leaves; note that the leaves were stored at room temperature in darkness or dim light for 2-3 days before the measurements (see Materials and Methods).

| Batch | RD±SD (Å) | n |
| --- | --- | --- |
| May  Low-light grown  High-light grown | 212±1  214±2 | 2  2 |
| March  Low-light grown  High-light grown | 220±1  223±1 | 2  3 |

**References**

Jakubauskas, D., Kowalewska, Ł., Sokolova, A. V., Garvey, C. J., Mortensen, K., Jensen, P. E. and Kirkensgaard, J. J. K. (2019). Ultrastructural modeling of small angle scattering from photosynthetic membranes. *Scientific Reports* **9**(1): 19405.

Ünnep, R., Zsiros, O., Solymosi, K., Kovács, L., Lambrev, P., Tóth, T., Schweins, R., Posselt, D., Székely, N. K., Rosta, L., Nagy, G. and Garab, G. (2014). The ultrastructure and flexibility of thylakoid membranes in leaves and isolated chloroplasts as revealed by small-angle neutron scattering. *Biochim Biophys Acta - Bioenerg* **1837**(9): 1572-1580.
